# Controlled Intestinal Microbiota Colonisation in Broilers under the Industrial Production System

**DOI:** 10.3390/ani12233296

**Published:** 2022-11-25

**Authors:** Advait Kayal, Dragana Stanley, Anita Radovanovic, Darwin Horyanto, Thi Thu Hao Van, Yadav S. Bajagai

**Affiliations:** 1 Institute for Future Farming Systems, Central Queensland University, Rockhampton, QLD 4702, Australia; 2 Faculty of Veterinary Medicine, University of Belgrade, 11000 Belgrade, Serbia; 3 School of Science, RMIT University, Bundoora, VIC 3083, Australia

**Keywords:** chicken, intestinal, colonisation, microbiota, Aviguard

## Abstract

**Simple Summary:**

Immediately post-hatch, young chicks are exposed to microbes in the air, feed and environment, and rapid colonisation of the gut begins. In environments loaded with pathogens, this process is critical with lifelong implications for the birds. Here, we present the large-scale commercial hatchery-based experiment using the “Hen in the bag” approach similar to faecal transfer in humans, where the highly diverse product, based on chicken caecal microbiota, was administered via automated spray equipment to the birds immediately post-hatch to mimic maternal inoculation. We report highly significant alterations in gut microbiota across upper and lower gut sections, changes in diversity in the caecum and jejunal mucosa, high resemblance of the inoculum microbial community to the caecal microbiota of the birds and consistently higher weight of treated animals.

**Abstract:**

The concept of designer microbiota in chicken is focused on early exposure of the hatchlings to pathogen-free microbiota inoculum, limiting the early access to harmful and pathogenic microorganisms, thus promoting colonisation of the gut with beneficial and natural poultry microbiota. In this study, we controlled colonisation of the intestine in broiler chickens in a large-scale industrial setting via at-hatch administration of a commercial product containing a highly diverse microbiota originating from the chicken caecum. The treatment significantly transformed the microbiota membership in the crop, proventriculus, jejunum and caecum and significantly altered the taxa abundance in the jejunum, jejunum mucosa, and caecum estimated using PERMANOVA and unweighted and weighted UniFrac distances, respectively. The treatment also improved the growth rate in chickens with no significant alteration in feed conversion ratio. A comparison of inoculum product microbiota structure revealed that the inoculum had the highest Shannon diversity index compared to all investigated gut sections, and the number of Observed Species second only to the caecal community. PCoA plots using weighted or unweighted UniFrac placed the inoculum samples together with the samples from the caecal origin.

## 1. Introduction

Broiler chickens represent a substantial part of the poultry industry, providing the affordable source of animal protein for the growing world population. The demand for chicken meat is rising due to its low environmental footprint and cost [1]. Approximately 20.4 million metric tons of broiler meat are produced in the United States, and about 14.7 million tons by the world’s second biggest producer China [2]. Australian poultry meat production was valued at approximately AUD 2.9 billion, according to a survey conducted in 2019–2020 [3]. The global poultry industry faces many challenges related to food safety and bird welfare, mainly brought by the switch to open and free-range production systems. The industry is continually investing in research to improve bird performance, food safety and meat quality while considering animal welfare.

The chicken intestinal microbiota is responsible for various physiological and metabolic processes necessary to maintain good health and productivity [4]. In addition, chicken microbiota mediates the response to stresses like heat [5], impacts immune response [6] and, via gut-brain axes, alters bird behaviour [7]. One of the major objectives of modifying intestinal microbiota is to increase the number of beneficial and reduce the number of pathogenic or harmful microbes. In addition to causing diseases, the presence of pathogens can compromise food safety which represents a major issue the industry is continually addressing to make the products safer for human consumption [8,9,10]. Supplementing the birds with probiotics to improve the ratio of beneficial to pathogenic intestinal bacteria can reduce enteric diseases like necrotic enteritis [11].

Studies show that the recent expansion and the industrialisation of poultry production systems affect the gut microbiota of the birds [12]. In the poultry industry, fertilised eggs are separated from the mother hen and incubated in a clean hatchery environment. This prevents the exchange of maternal microbiota between the mother hen and the chick [12]. From the hatcheries, the newborn chicks are transported directly to the farm. During the transportation, the chicks can acquire poultry uncharacteristic microbiota from the trucks, the environment or the humans [12]. This leads to poor microbiota reproducibility, with different batches of birds originating from the same breeding stock and hatchery, raised on the same batch of food and in the same shed, demonstrating massive differences in microbial community as high as at a phylum level [12]. Since microbiota plays a major role in health, immune response, behaviour and performance [13], poor microbiota reproducibility results in a different response to stresses, pathogens, and environment, ultimately leading to variable flock performance [12].

Probiotic products are among the most widely used poultry supplements. However, it is accepted that most probiotics rarely colonise and persist in the gut and that continual supplementation is needed [14]. As our understanding of the role of microbiota in the birds’ health and productivity advanced in recent years, novel ways of administering probiotics to ensure persistence in the gut are being explored. Some probiotics can be administered in-ovo, via injection into the egg amniotic fluid [15], others are designed to be sprayed onto the hatchlings before transport, while the majority of products are conventionally regularly administered into feed and very few in drinking water [16]. Environmental factors play a major role in gut colonisation. *Campylobacter jejuni* is a known commensal that starts colonisation due to the various short chain fatty acids (SCFAs) produced by the existing gut microbiota [17]. A form of mutualism can be observed with *Lactobacillus salivarius* strains as they acquire different genes responsible for energy and nutrient utilisation depending on animal hosts [18]. Competitive exclusion due to the limited availability of nutrients can also affect colonisation and prevent pathogens from colonising in the chicken gut [19]. Additionally, nutrient landscape determines successful colonisation and the ability of microbes to persist long term. In this highly competitive environment, the isolates capable of efficiently using limiting nutrients will have colonisation advantage [20].

The administration of probiotics can affect the histomorphology of the intestinal tract. The goblet cells are essential in producing mucin and maintaining intestinal homeostasis by providing bicarbonate [21,22]. These cells also play a role in the immune system as a line of defence at the mucosal layer and deliver soluble antigens in the intestine to underlying dendritic cells [23]. This function can prevent the colonisation of pathogens in the gut and help maintain the gut health of the chickens.

Designer microbiota is a recent concept that aims to control the exposure of the intestine to the predesigned and reproducible microbial population, restricting early access to pathogens and allowing the beneficial microbes to colonise the gut. Wilkinson et al. [24] showed that controlled gut colonisation is possible immediately post-hatch in a controlled environment. In contrast to the controlled colonisation that requires a challenging process of generation of precisely defined and reproducible community, faecal transplant commonly performed in humans focuses on the health and performance of the donor, accepting the degree of individual and temporal microbiota variation.

Our research hypothesis was that early intervention in chicken gut colonisation could bring beneficial modification in the gut microbiota population, which would eventually help to improve the growth rate and feed efficiency. This study aimed to investigate an alternative chicken gut colonisation intervention by administering a commercial product originating from chicken caeca with a highly diverse microbiota, similar to the procedure used for human faecal transplants. The inoculum was produced using biotechnology able to provide a highly reproducible microbial community.

## 2. Materials and Methods

### 2.1. Animal Trial

The poultry trial was conducted in a commercial hatchery and an associated large-scale broiler farm in New South Wales, Australia. The experiment was conducted with 164,000 Cobb-500 broiler chicks. Immediately after hatch, 82,000 chicks were placed in trays (100 chicks in each tray) and put through a spraying applicator over a conveyer belt. The chicks were sprayed with a microbiota inoculum (Aviguard^®^, Lallemand Animal Nutrition, Canada). The commercial, high throughput edible gel droplet delivery system (Gel-Pac, Animal Science Products Inc, Nacogdoches, TX, USA) was used at default settings. Gel-Pac was designed to rapidly deliver vaccines, prebiotics, phytogens, immune modulators and various medical treatments. More information about the Gel-Pac system is available on the web manual [25]. The inoculum was diluted at the recommended dose and mixed with green gel food dye, so visible green droplets, 1–3 mm in size, were formed on birds encouraging them to preen the liquid from one another. The droplets were ingested quickly; within 2–5 min, as estimated by the disappearance of the green droplets on the birds. The tongues of the birds were randomly checked to confirm that they were green, as recommended in the manual [25].

The remaining 82,000 chicks, used as the control, were sprayed with water mixed with gel dye without the microbiota inoculum. Hatchery conditions were maintained at 25 °C and relative humidity around 55%. Control (CTR) and Aviguard treatment (AVG) groups were physically separated during the spray and transported to the growing farm in separate trucks.

The birds were reared in four temperature-controlled sheds with 41,000 birds in each. The four sheds were adjacent, with a minimum 20 m space between them. The sheds had controlled heating and operated as a barn type, without access to the outdoors. The management practices in all four sheds were the same. A regular commercial diet meeting the nutritional requirement recommended for the breed, and water were provided ad libitum. The birds were picked up from the shed for processing in batches from day 32 to day 54. The farm veterinarian provided the average final body weight and feed conversion data. The feed conversion ratio (FCR) was calculated by dividing the total amount of feed consumed by the total live weight sold. As the birds were sold in batches on different days, the FCR was adjusted for 2.45 kg as reference body weight in both the control and treatment groups to make it comparable. The adjusted FCR (cFCR) was calculated by multiplying the differences in actual body weight and reference body weight with the correction factor of 0.20 and adding to the actual FCR.

The poultry experiment was approved by the Animal Ethics Committee of Central Queensland University (Approval number 0000023123).

### 2.2. Sample Collection

At the age of 28 days, ten randomly selected birds from each shed were euthanised, dead weight was collected using a hanging scale, and jejunum, caecal and crop content were collected. Jejunal content and jejunal mucosal swab samples were collected from around the mid-section between the posterior end of the duodenal loop and Meckel’s diverticulum. Crop content was collected by making the lateral incision and exposing the crop content. A large quantity of crop content was collected and homogenised prior to DNA extraction. Mucosal swab samples were also collected from the proventriculus mucosa. To prevent cross-contamination, gut sections were separated into disposable trays before sample collection. All field-collected samples for DNA extraction were stored in the Nucleic Acid Preservation (NAP) buffer described by Menke and coauthors [26]. The samples were kept in ice during the collection and transport and stored at −80 °C until processing. Three random samples of the commercial product Aviguard (AVG powder) were sequenced using the same 16S sequencing methodology as the intestinal microbiota samples.

We also sampled birds on days 1, 3, and 5, in order to observe the temporal aspect of gut development, but the size of the gut sections was very small, so we could not get enough sample for DNA extraction, and many of the samples would not amplify; therefore, these samples were excluded from the study.

### 2.3. DNA Extraction, Sequencing and Data Analysis

The genomic DNA from the samples were isolated using the lysis protocol developed by Yu and Morrison [27] and purified using DNA mini spin column (Enzymax LLC, CAT# EZC101, Lexington, KY, US). The quantity and quality of the DNA were measured using NanoDrop One UV-Vis spectrophotometer (ThermoFisher Scientific, Waltham, MA, USA).

The V3-V4 region of the 16S rRNA gene was amplified using the following primers with spacers, barcodes, and Illumina sequencing linkers [28]. The forward primer was 338F (5′-ACTCCTACGGGAGGCAGCAG-3′) and the reverse primer was 806R (5′-GGACTACHVGGGTWTCTAAT-3′). The resultant 16S amplicon library was purified by using AMPure XP kits (Beckman Coulter, Brea, CA, USA) and sequenced with the Illumina MiSeq platform with 2 × 300 bp paired-end configuration. The read with better quality was used downstream with a minimum Phred score of 20 across the length of 200 nt. Raw DNA sequences were demultiplexed with Cutadapt [29] and analysed with Quantitative Insights into Microbial Ecology 2 (QIIME 2) [30] using DADA2 [31] for filtering, denoising and chimaera removal. DADA2 was also used to trim the reads based on the parameters selected from the QIIME 2 sequence quality control parameters. SILVA v 138.1 database [32,33] was used as a reference to assign taxonomy. The ASV data were clustered into OTUs at 98% similarity. Only the part of the analysis attempting to predict colonisation success was done at an ASV level, while all the remaining data were analysed at an OTU level. The analysis and interpretation of the data were completed through the data rarefied at a minimum of 3000 sequences per sample. R packages, including Phyloseq, Phylosmith, Vegan and Microeco were used for further downstream analysis and visualisation of the data. The raw sequence data has been uploaded to NCBI SRA database with accession number PRJNA887826.

### 2.4. Histology

The samples for histomorphology were collected from the jejunum. The samples were fixed in 10% neutral buffered formalin solution. The further processing was outsourced to the Veterinary Laboratory Services at The University of Queensland, Gatton, Australia. The tissue processing involved fixation, paraffin embedding and microtoming. The embedded samples were cut to 4 μm thickness using LEICA RM2135 microtome (Leica Biosystems, Wetzlar, Germany). These slides were stained using the Periodic Acid-Schiff-Alcian Blue staining method. The slides were scanned using Panoptiq™ software (ViewsIQ Inc., Vancouver, BC, Canada) and Nikon Eclipse Ci-L Plus biological microscope (Nikon Corporation, Minato-ku, Japan). Villus height, crypt depth, villus width, villus area and the number of goblet cells were measured from 10 randomly selected well-positioned villi per slide and six slides per group, three from each shed.

### 2.5. Statistical Methods

Mann–Whitney test performed in GraphPad Prizm 9 was used to compare the animal weights, alpha diversity and histological measurements. Alpha diversity indicators were previously calculated using Phyloseq R package. Distance matrices (UniFrac and Bray–Curtis) were calculated from the rooted Newick OTU tree (the tree was obtained in QIIME 2), in the Microeco R package, which was also used to calculate all beta diversity, including PCoA and PERMANOVA. Metastats function for univariate analysis was performed in Microeco and plotted in GraphPad Prizm 9.

## 3. Results

### 3.1. Bird Performance

**Figure 1:**
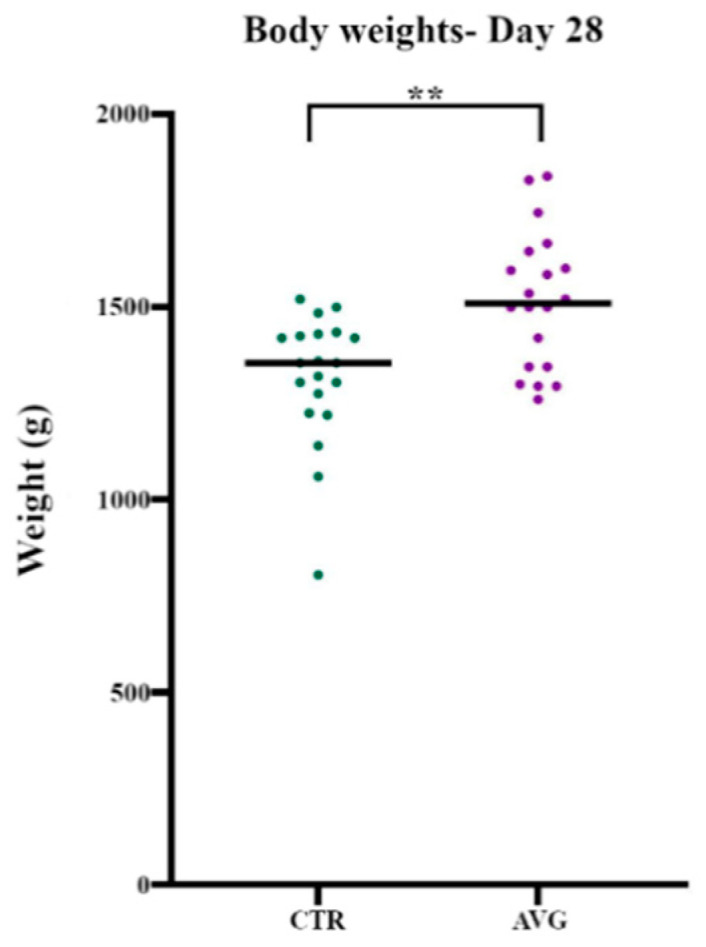
Body weights of birds on day 28 (Sample collection day). ** represent *p =* 0.0026.

The data collected from birds euthanised on day 28 (Table 1) showed that the average body weight of Aviguard treated birds (1516 g) was significantly higher (*p =* 0.0026) than the body weight of control birds (1318 g) (Figure 1). Similarly, the final average body weight data for the birds that were sold to the market showed that the average body weight of Aviguard treated birds (3070 g) was higher than the average body weight of control birds (2760 g). The FCR in Aviguard sheds (1.779) was marginally higher than the FCR in control sheds (1.741), while cFCR, adjusted for 2.45 kg body weight as described above, was 1.678 for control and 1.656 for Aviguard.

### 3.2. Community Structure

**Figure 2:**
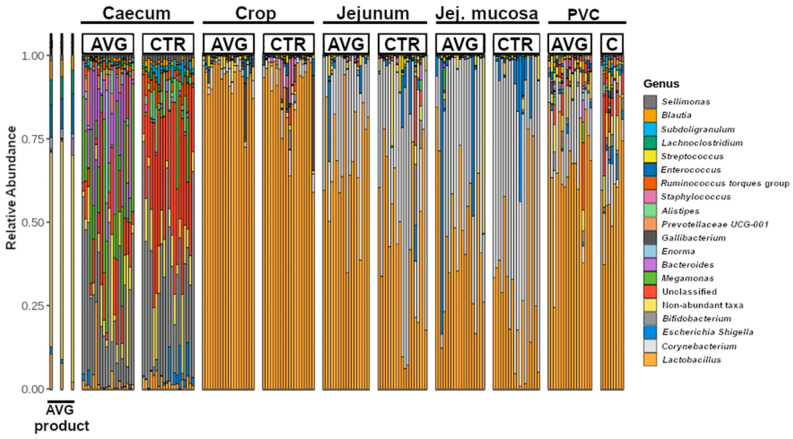
Genus-level relative abundance, including the twenty most abundant genera. The plot shows only known genera, while all unknown and unclassified genera from all families were binned into “Unclassified”. Jej.mucosa = Jejunum mucosa, PVC = proventriculus.

The broiler microbial communities collected from the caecum, crop, jejunum, jejunal mucosa and proventriculus mucosa were dominated by sequences assigned to phyla *Firmicutes* and *Actinobacteria*, followed by *Bacteroidota* in the caecum and *Proteobacteria* in other sections. Lower abundant phyla included *Fusobacteriota*, *Campylobacteriota*, *Desulfobacterota*, *Verrucomicrobiota*, *Acidobacteriota* and *Chlorofexi*. With a visible distinction of genus level membership in the caecal community (Figure 2), the major dominating genera were *Lactobacillus*, *Corynebacterium*, *Escherichia-Shigella*, *Bifidobacterium*, *Megamonas*, *Bacteroides*, *Enorma*, *Gallibacterium*, *Alistipes*, *Streptococcus*, and *Staphylococcus*.

Aviguard is a reproducible chicken caecal community comprised of multiple non-pathogenic species typically present in the most diverse gut section—caecum. The presence of non-pathogenic strains of species that can contain major pathogens employs the mechanisms of competitive exclusion to prevent or reduce colonisation with pathogenic strains. The major genera we identified in the Aviguard product are *Enterococcus*, *Lachnoclostridium*, *Negativicoccus*, *Peptostreptococcus*, *Clostridium*, *Lactobacillus*, *Haloimpatiens*, *Blautia*, *Eubacterium*, *Enorma* and *Megasphaera*, while *Fusobacterium*, *Slackia*, *Bacteroides*, *Flavonifractor*, *Collinsella*, *Paraclostridium*, *Sutterella*, *Escherichia-Shigella*, *Sellimonas*, *Butyricicoccus*, *Erysipelatoclostridium*, *Candidatus*, *Olsenella* and *Megamonas* were present in lower abundance based on sequence number. There were also species belonging to unknown genera from *Ruminococcaceae*, *Lachnospiraceae* and *Prevotellaceae*.

### 3.3. Alpha and Beta Diversity

**Figure 3:**
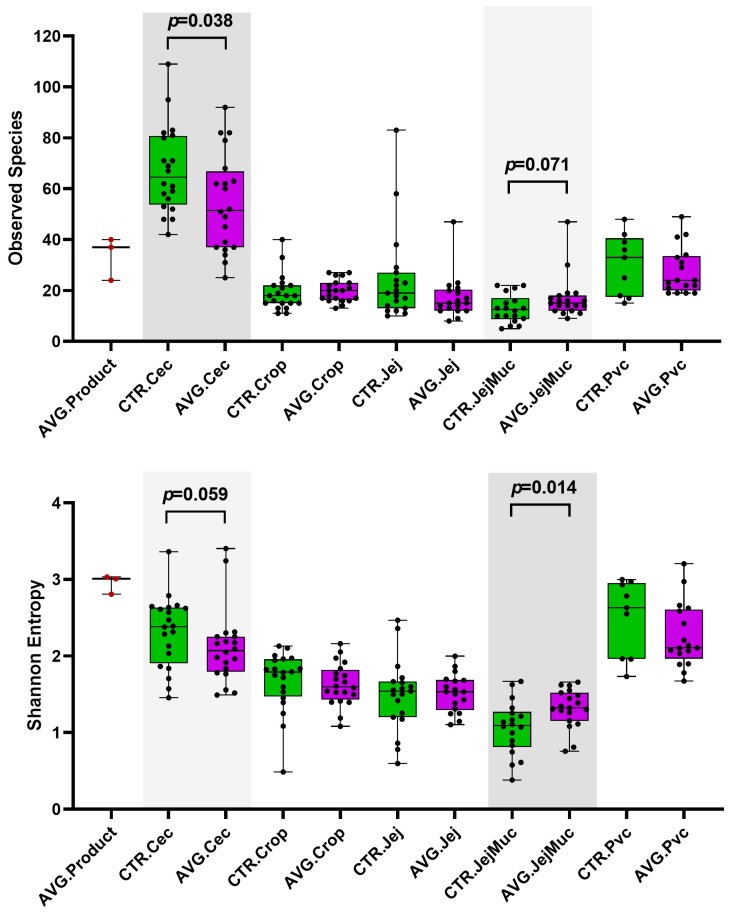
Alpha Diversity plots showing the Observed Species and Shannon Entropy. Cec = caecum, Jej = jejunum, Jej.Muc = jejunal mucosa, Pvc = proventriculus.

Alpha and beta diversity were investigated to compare the microbial communities between AVG-treated and untreated groups in the different intestinal sections. Caecum samples exhibited a significantly higher richness than other sections, measured with observed species. Proventriculus and caecum residing microbial communities had higher diversity than other sections, as assessed by the Shannon entropy index (Figure 3). Shannon diversity index showed that AVG product contains a highly diverse microbiota population. The jejunum mucosal samples had the lowest richness and diversity values, but the AVG-treated group was significantly higher than the control group.

**Figure 4:**
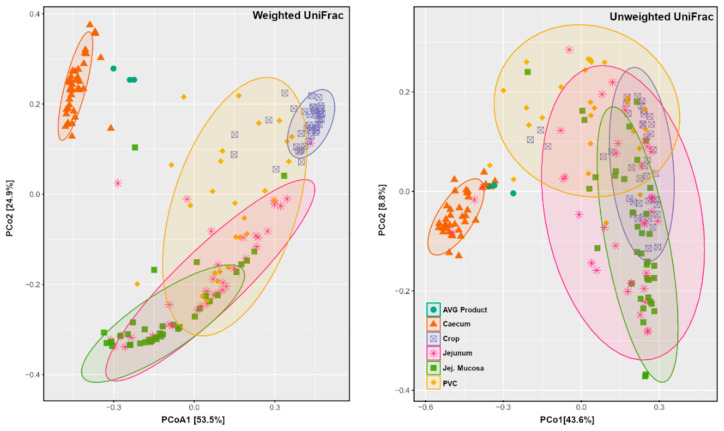
PCoA plots generated using weighted and unweighted UniFrac distance. Jej.Mucosa = Jejunal Mucosa, PVC = Proventriculus.

The Principal Coordinate Analysis (PCoA) ordination of weighted and unweighted UniFrac distances depicted that AVG is ecologically more similar to caecum samples than other sample types (Figure 4). The PCoA plots demonstrate the clear distinction between caecal and AVG product samples from other sections of the gut. Among other sections, the tight grouping and separation of crop samples for weighted UniFrac distance but not for unweighted UniFrac distance indicated no difference in crop bacteria membership but rather a different abundance distribution among crop samples.

To analyse if AVG introduced significant microbiota alterations in any of the gut sections, we used Permutational Multivariate Analysis of Variance (PERMANOVA) for main variables and Paired Multivariate Analysis of Variance (MANOVA) for paired comparisons (Table 2), at both weighted and unweighted UniFrac distances. Based on PERMANOVA, the Shed variable and origin (caecal, proventriculus, jejunum content and mucosa, and the crop) had a significant (*p* < 0.001) influence on microbiota using both weighted and unweighted UniFrac distance. Based on weighted UniFrac, Control and AVG differences were most prominent in the caecal (*p* < 0.001), followed by jejunal microbial communities and not significantly altered in the upper digestive tract communities of the crop or proventriculus. When observing presence data via unweighted UniFrac distance (Table 2), caecum microbiota was again the most affected by AVG treatment, followed by microbiota in proventriculus, jejunum and crop, while there were no significant changes in jejunal mucosa. Thus, AVG introduced drastic changes in the microbiota, predominantly in the caecum and, to a lesser but still significant level, in other sections of the gut in both membership and abundance.

### 3.4. Long Term Colonisation

We performed Venn diagram analysis to investigate OTUs and ASVs shared between the AVG product and all other groups in each origin. The data were inconclusive since the AVG product representative sequences using relatively small amplicon lengths are very common in poultry gut species; they were detected indiscriminately. The 16S short amplicon methodology does not have the resolution to separate the origin of the OTU without at least the full length of the 16S sequence or additional biomarkers. Since the PERMANOVA data from Table 2 by both weighted and unweighted UniFrac implicate caecum as the most affected gut origin, and, more convincingly, the PCoA plots (Figure 4) place AVG product samples with caecal microbiota of both AVG and CTR group.

**Figure 5:**
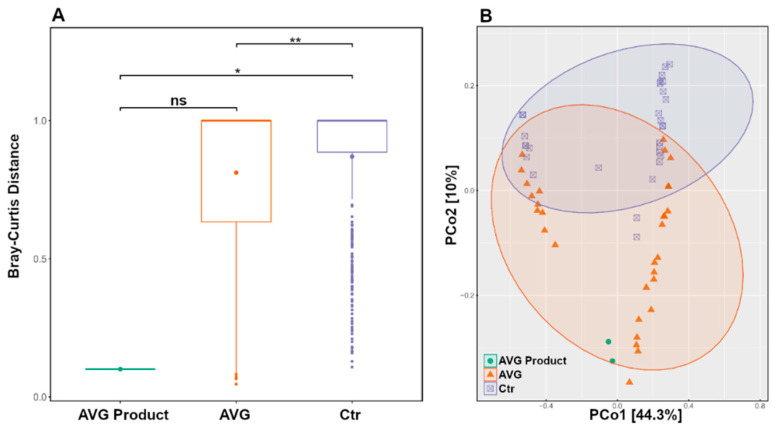
PERMANOVA boxplot (Panel (**A**)) and Unweighted UniFrac PCoA plot (Panel (**B**)) on caecal microbiota subset. (ns = not significant, ** *p* < 0.01; * *p* < 0.05). While the rest of the data in this manuscript was presented at an OTU level, this data was analysed at an ASV level.

Using only the caecal microbiota subset and ASV level data, the PERMANOVA box plot (Figure 5A) shows significant microbiota differences between CTR and AVG treated groups (*p* < 0.01) and CTR and AVG product (*p* < 0.05) while there is no significant difference between AVG group and AVG product in caecal microbiota. This is further confirmed by the unweighted UniFrac PCoA plot (Figure 5B), where the AVG product samples were the most similar to the microbiota of the caecal contents of AVG-treated birds and far separated from the microbiota of samples of the CTR birds in agreement with the boxplot (Figure 4).

### 3.5. Univariate Taxa Alterations

**Figure 6:**
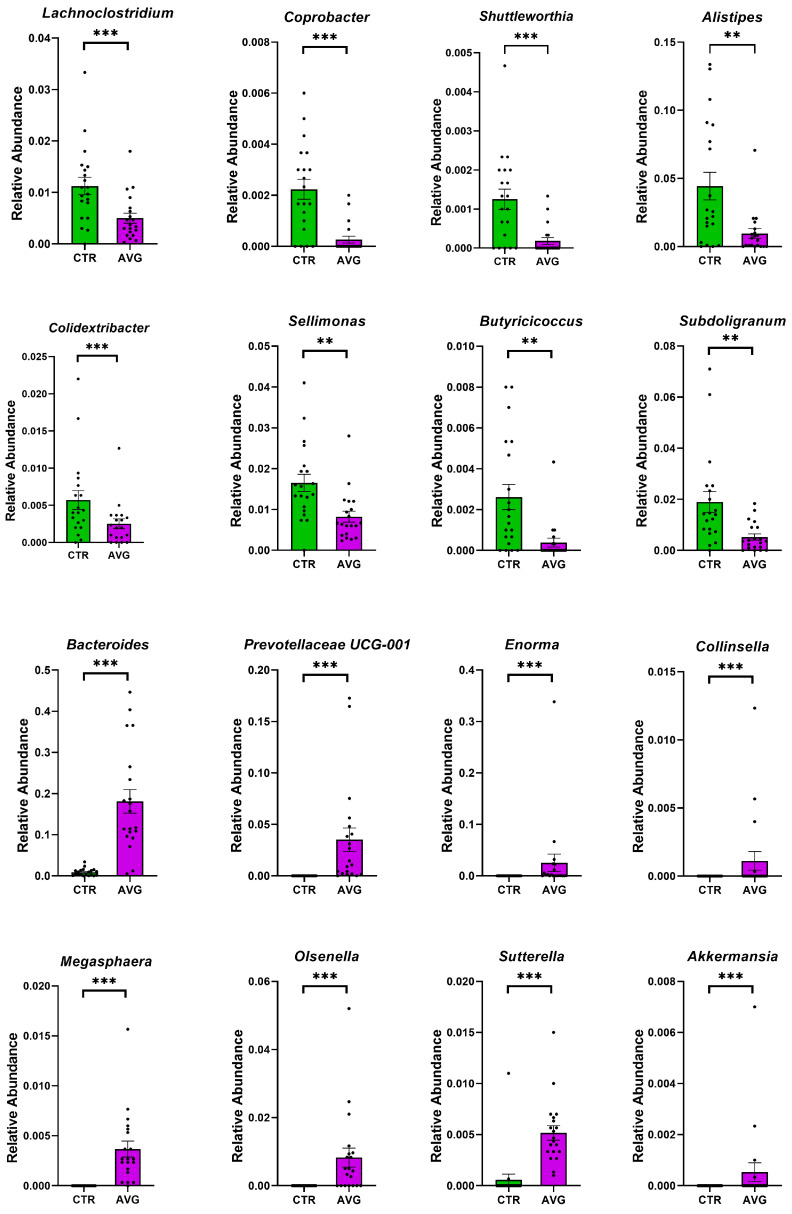
Metastats selected differentially (*p* < 0.001) abundant genera in the caecum. The asterisk indicates significance level (*** *p* < 0.001; ** *p* < 0.01). Each dot represents one sequenced sample relative abundance.

**Figure 7:**
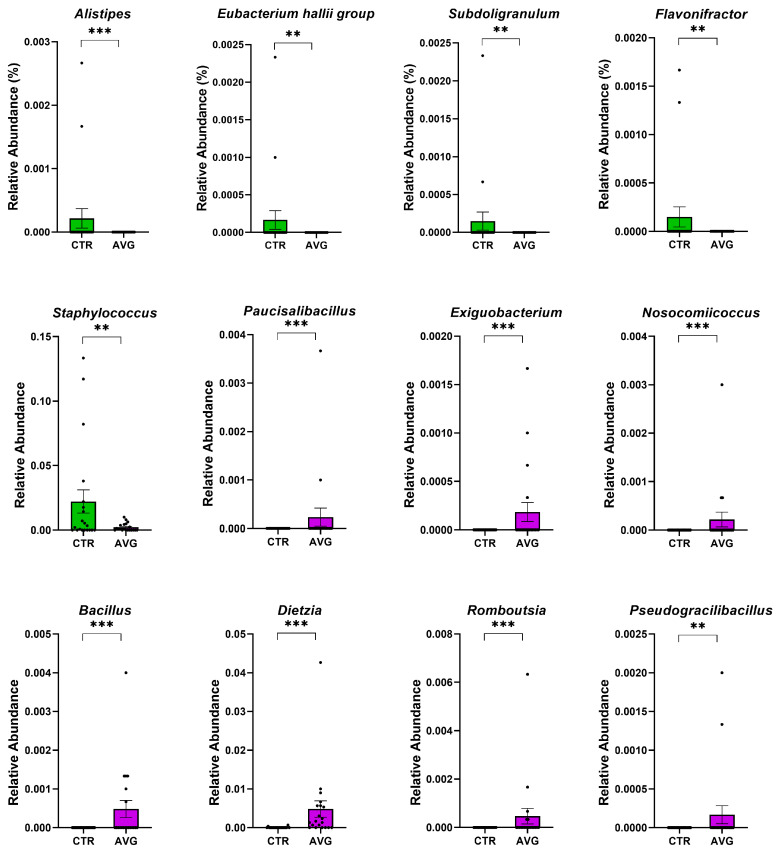
Metastats selected differentially (*p* < 0.001) abundant genera in the crop. The asterisk indicates significance level (*** *p* < 0.001; ** *p* < 0.01). Each dot represents one sequenced sample relative abundance.

**Figure 8:**
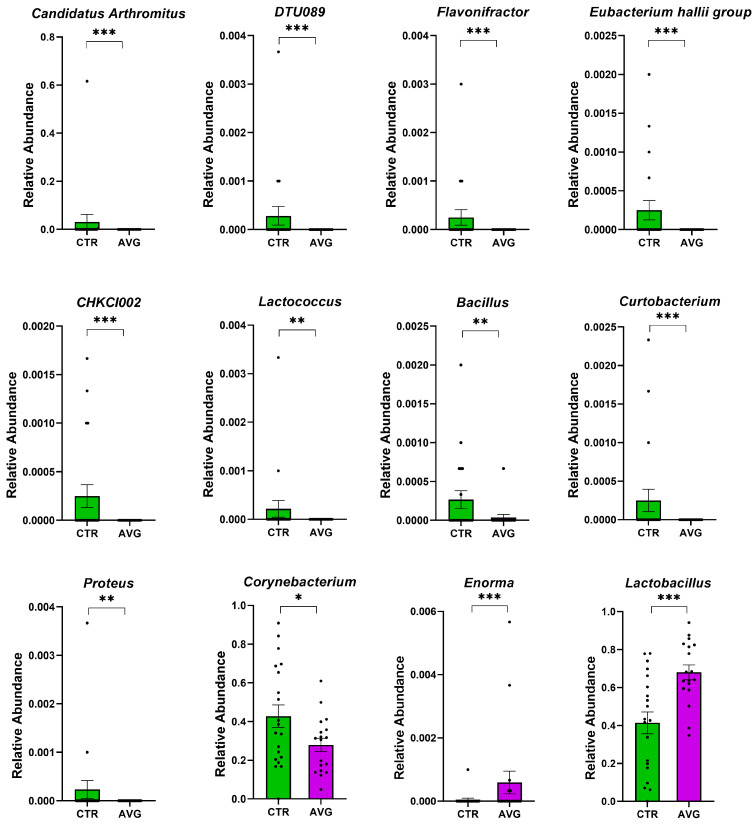
Metastats selected differentially (*p* < 0.001) abundant genera in the jejunum. The asterisk indicates significance level (*** *p* < 0.001; ** *p* < 0.01; * *p* < 0.05). Each dot represents one sequenced sample relative abundance.

**Figure 9:**
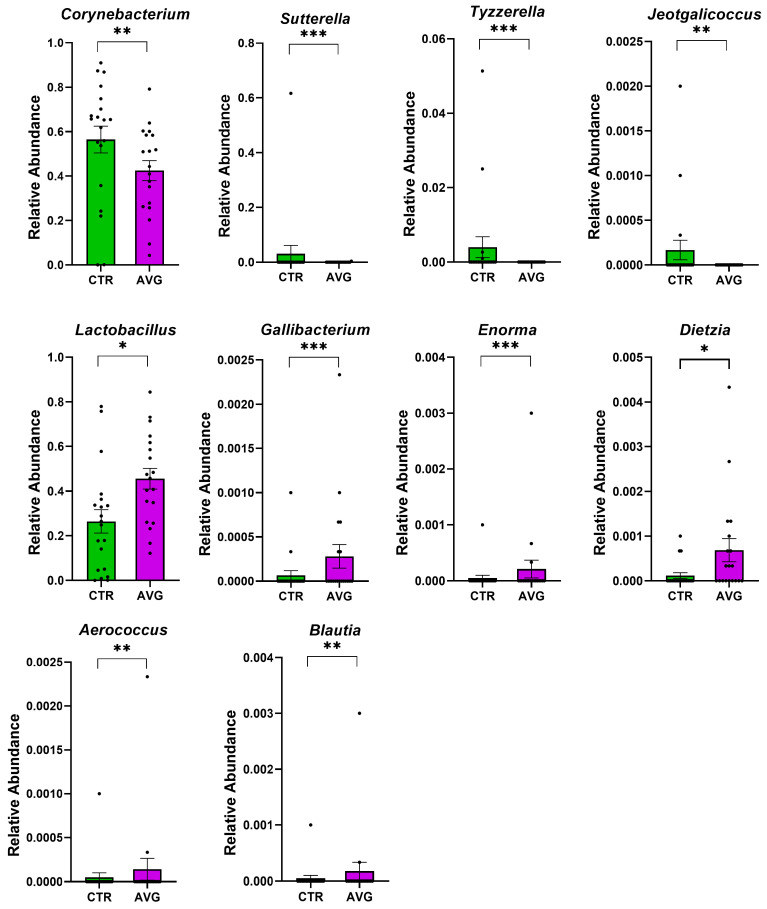
Metastats selected differentially (*p* < 0.001) abundant genera in the jejunal mucosa. The asterisk indicates significance level (*** *p* < 0.001; ** *p* < 0.01; * *p* < 0.05). Each dot represents one sequenced sample relative abundance.

We used Metastats [34] for differentially abundant feature analysis in each intestinal section. This method controls the false discovery rate and applies Fisher’s exact test, which is considered a suitable statistical method for sparsely sampled features as in microbiota study. All sections except the proventriculus were highly balanced in the number of samples kept in the analysis after the rarefaction: caecum (20 AVG vs. 20 CTR), crop (20 AVG vs. 20 CTR), jejunum (18 AVG vs. 19 CTR), jejunal mucosa (19 AVG vs. 18 CTR) and proventriculus (17 AVG vs. 9 CTR). Due to a high number of proventriculus swabs failing in either PCR amplification or the number of sequences per sample filtering, we used this gut section in figures to graphically present alpha and beta diversity and in PERMANOVA comparisons that can deal with imbalanced data, but this section was not used for differential abundance analysis. The selected features differential between the AVG and the control are presented in Figure 6 (caecum), Figure 7 (crop), Figure 8 (jejunum) and Figure 9 (jejunal mucosa).

In the caecum samples, the most differential genera were *Lachnoclostridium*, *Coprebacter*, *Alistipes*, *Colidextribacter*, *Butirycicoccus*, *Bacteroides*, *Provotellaceae UCG-001*, *Enorma*, *Megasphaera* and *Olsenella*. Some of the most differential genera in the crop content samples were *Alistipes*, *Eubacterium hallii* group, *Subdoligranulum*, *Flavonifactor*, *Staphylococcus*, *Bacillus*, *Dietzia*, *Exiguobacterium* and *Romboutsia*. The differential genera found in the jejunum samples included *Flavonifactor*, *Eubacterium hallii* group, *Lactococcus*, *Bacillus*, *Corynebacterium*, *Enorma* and *Lactobacillus*. *Corynebacterium*, *Sutterella*, *Lactobacillus*, *Gallibacterium*, *Enorma* and *Dietzia* were some of the most differential genera in the jejunum mucosa.

### 3.6. Histology

**Figure 10:**
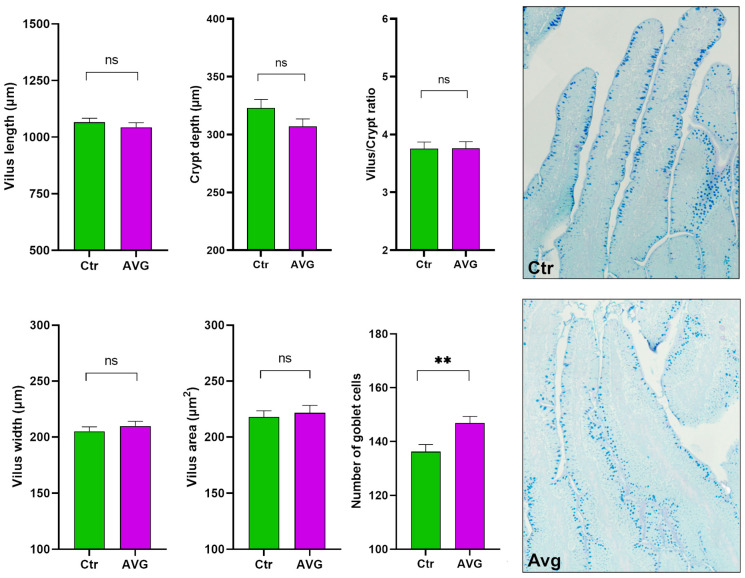
Histological measurements of jejunum. AVG has significantly more goblet cells. The goblet cells in AVG were smaller and noticeably more abundant in the crypt region. Both AVG and Ctr histological images are given at 20× magnification. Significance (ns = not significant; ** *p* < 0.01).

There were no significant differences in villus height (Mann–Whitney test *p* = 0.12), villus width (*p* = 0.28), villi area (*p* = 0.79), crypt depth (*p* = 0.22), or villus/crypt ratio (*p* = 0.98) (Figure 10). These results indicate that measured parameters remained unaffected by Aviguard supplementation. The number of goblet cells was significantly higher (*p* = 0.0021) in the Aviguard treated group compared to the control (Figure 10), and this increase is more noticeable in the crypt than in the region of the villi. Mucin granules in the goblet cells demonstrate mostly a light blue colouration in both experimental groups, indicating that epithelium goblet cells in jejunum might contain only acidic mucin. (Figure 10).

## 4. Discussion

Designer gut microbiota is a recent concept that involves controlling the gut’s exposure to microorganisms to obtain more uniform and beneficial gut microbiota. Different methods have been attempted for gut manipulation and creating designer microbiota. Early gut intervention can cause long-term and stable alterations in the bacterial and metabolic composition of the gut [35], which in turn can also alter the gene expression in the host [36] and improve immunity against enteric pathogens [37]. Moreover, early intervention and controlled colonisation can also reduce the relative abundance of pathogens such as *Enterococcus* and *Escherichia-Shigella* and increase the concentration of short-chain fatty acids (SCFAs) like acetate, propionate, butyrate and isovalerate, thus improving gut health [38]. Controlled colonisation with a faecal transplant can also influence intestinal histomorphology [39] and growth rate [35,39].

Aviguard significantly affected the alpha diversity in the caecum and jejunum mucosa microbial communities. Both richness, as indicated by observed species, and diversity measured with Shannon entropy decreased in the Aviguard group’s caecum. Contrary to our results, a recent study has shown that caecal microbiota transplants increase the richness and diversity of caecal microbiota in treated birds [40]. Samples in this caecal microbiota transplant study were collected from day 1 to day 7 after the transplant, in contrast to our study, where samples were collected much later at day 28 as we aimed to investigate long-term effects of Avigurad at the end of the production cycle. Unlike in caecum, the richness and diversity were increased in the jejunal mucosa microbiota. Other gut sections were also marginally affected. Diversity in the caecum microbiota, representing the most diverse section of the gut, was reduced, while it was increased in jejunal mucosa, one of the least diverse microbial communities. This increase in jejunal mucosa microbial diversity could have been influenced by the early arrival of AVG product species into the naïve gut of treated chicks. The jejunum mucosal layer is more challenging to colonise as it supports highly specialised microbial community membership [41]. This could be associated with genetic resistance [42,43] or expression of an immunogene [44] as observed against pathogens like *Salmonella* [42,43] and *Campylobacter jejuni* [44]. The increase in the mucosal diversity could be attributed to AVG Product containing more species capable of utilising, degrading and/or moving through the mucous layer, leading to colonisation with non-pathogenic taxa and competitive exclusion of pathogens.

The beta diversity analysis showed that administration of Aviguard significantly affected the weighted UniFrac distance in the caecum, jejunum and jejunum mucosa and significantly affected the unweighted UniFrac distance in all the gut sections except jejunum mucosa. The weighted UniFrac distance measures the phylogenetic relationship between samples considering the abundance of individual taxa while unweighted UniFrac distance shows the phylogenetic relationship based on the presence or absence (membership) of taxa. Further investigation showed no significant differences in Bray–Curtis distance between the AVG product and the caecum content of the AVG treated group. This indicated that microbiota in AVG products is ecologically more similar to the caecum of AVG treated birds than to the caecum of CTR birds. These observations suggest that AVG likely colonised caecum to some extent. However, the significant alteration of overall caecal microbiota by AVG administration does not necessarily indicate that many AVG species permanently colonised the caecum. Even a single species arriving at the caecum early in the process of colonisation could alter the colonisation to result in different final abundance and membership. This logic also applies to other sections.

Table 2 shows that the upper sections of the digestive system, crop and proventriculus, were not altered in abundance (weighted UniFrac). The upper gut would be at the forefront of AVG exposure which can explain the significant alteration in the presence and absence of different taxa. Although it is challenging to investigate the influence of AVG on the temporal dynamics of colonisation due to the complexity and richness of the product, we must acknowledge the number of other variables that can affect and interfere with the colonisation process, one of the most important being environmental and feed microbial community. The differences in these background communities can be rather dramatic, especially in the sheds that experience frequent disease outbreaks. However, adverse effects are implausible since AVG product does not contain poultry pathogens. In challenging environments, the administration of AVG can ensure that benign and beneficial commensal species from the product outcompete pathogens through competitive exclusion during the early colonisation process.

Aviguard increased the abundance of several microbial genera in different gut sections. The bacterial genera enriched in the Aviguard group, reported previously as beneficial SCFA producers, were *Lactobacillus* [45,46,47], *Flavonifractor* [48], *Megasphaera* [49], *Bacteroides* and *Blautia* [50]. Similarly, the genera linked to better growth performance, improved immune response, better intestinal morphology, increased nutrient digestibility and improved energy metabolism, such as *Bacillus* spp. [51,52,53,54], *Dietzia* [55], *Romboutsia* [56], *Sutterella* [57], *Lactococcus* [58,59] and *Olsenella* [60], had a higher abundance in the AVG treatment group.

Aviguard reduced the abundance of some common bacterial genera containing pathogenic species like *Staphylococcus*, *Proteus* [61,62], while *Gallibacterium* [63] was increased. Some beneficial genera associated with improved growth, such as *Butyricicoccus*, *Subdoligranulum* [50], *Alistipes* [64], *Lachnoclostridium* and *Shuttleworthia*, were reduced in the Aviguard group. Nevertheless, the above beneficial or pathogenic taxa effects on the birds cannot be speculated based on 16S microbiota data because this methodology cannot distinguish taxa at the species level; and both beneficial and pathogenic effects are highly strain-specific.

Although Aviguard did not significantly alter the villi heights, width, crypt depth, height to depth ratio and villi area, there was a significant increase in the number of goblet cells. This is a beneficial effect of Aviguard as these cells produce mucin [21,22] and can also be instrumental in protecting the gut against pathogens [23]. Further studies are required to understand why the goblet cells were majorly increased in the crypt region and to understand the importance of this finding.

## 5. Conclusions

Performing large-scale studies under industrial conditions is an excellent way to reproduce the actual conditions endured by animals. However, industrial trials often lack precision in performance measures collection and near complete control of variables involved that is present in studies done in highly controlled experimental animal facilities. On the other hand, while providing a statistically superior setup, perfectly controlled trials in animal research facilities often present conditions very far from real production. Investigating the controlled colonisation and designer microbiota concept will require combining both approaches. Here, we presented the large-scale application of AVG in commercial hatcheries, demonstrating that the product at-hatch administration was highly automated and simple. Our data agree with the AVG inoculum’s caecal origin and its exceptionally high diversity. We also report that AVG administration resulted in significant differences in all gut sections concluding that AVG strongly influenced the dynamics of the colonisation process. However, using the 16S methodology does not allow us to speculate on the permanent colonisation of different species. More controlled and industry-scale experiments are needed to dissect the influence of background microbiota in the feed, shed and hatchery on the reproducibility of the colonisation alterations by AVG. Shotgun metagenomics data would help investigate the transfer of functional capabilities and would likely provide evidence of the species transfer from AVG product into the birds.

## Figures and Tables

**Table 1:** Average body weight of the birds on day 28 (Sample collection day).

Group	Average Body Weight (g)	SEM
Control	1318	37.9
AVG	1516	39.5

**Table 2:** PAIRED MANOVA effects of AVG on intestinal microbiota of different gut origins.

Measures	Groups	R2	*p* Value	Significance
Weighted UniFrac	Cec.AVG vs. Cec.CTR	0.237666	<0.001	***
	Jej.AVG vs. Jej.CTR	0.150488	0.007	**
	JejMuc.AVG vs. JejMuc.CTR	0.101046	0.023	*
	Crop.AVG vs. Crop.CTR	0.029126	0.32	
	PVC.AVG vs. PVC.CTR	0.043081	0.353	
Unweighted UniFrac	Cec.AVG vs. Cec.CTR	0.188473	<0.001	***
	PVC.AVG vs. PVC.CTR	0.11597	0.005	**
	Jej.AVG vs. Jej.CTR	0.068658	0.01	**
	Crop.AVG vs. Crop.CTR	0.062496	0.014	*
	JejMuc.AVG vs. JejMuc.CTR	0.038949	0.163	

* Cec = Caecum; Jej = Jejunum, JejMuc = Jejunal Mucosa, PVC = Proventriculus. Significance (*** *p* < 0.001; ** *p* < 0.01; * *p* < 0.05).

## Data Availability

The raw sequence data is available from NCBI SRA database with accession number PRJNA887826 (https://www.ncbi.nlm.nih.gov/bioproject/887826).
